# Examining the links between community participation and health outcomes: a review of the literature

**DOI:** 10.1093/heapol/czu076

**Published:** 2014-09-11

**Authors:** Susan B. Rifkin

**Affiliations:** Colorado School of Public Health, Denver Colorado USA.

**Keywords:** Community participation, evidence-based policy, health care reform, health outcomes

## Abstract

As a key principle of Primary Health Care (PHC) and Health Systems Reform, community participation has a prominent place in the current global dialogue. Participation is not only promoted in the context of provision and utilization of health services. Advocates also highlight participation as a key factor in the wider context of the importance of social determinants of health and health as a human right. However, the evidence that directly links community participation to improved health status is not strong. Its absence continues to be a barrier for governments, funding agencies and health professionals to promote community participation. The purpose of this article is to review research seeking to link community participation with improved health status outcomes programmes. It updates a review undertaken by the author in 2009. The search includes published articles in the English language and examines the evidence of in the context of health care delivery including services and promotion where health professionals have defined the community’s role. The results show that in most studies community participation is defined as the intervention seeking to identify a direct causal link between participation and improved health status modeled on Randomized Control studies (RCT). The majority of studies show it is not possible to examine the link because there is no standard definition of ‘community’ and ‘participation’. Where links are found, they are situation-specific and are unpredictable and not generalizable. In the discussion, an alternative research framework is proposed arguing that community participation is better understood as a process. Once concrete interventions are identified (i.e. improved birth outcomes) then the processes producing improved health status outcomes can be examined. These processes may include and can lead to community uptake, ownership and sustainability for health improvements. However, more research is needed to ensure their validity.

**KEY MESSAGES**

- There is a body of literature examining health improvements for poor populations that highlights community participation as a key factor.
- Evidence, most often investigated in the context of Random Case Control Trials (RCT) which seeks generalizable results and where community participation has been defined as an intervention, has proved illusive.
- A review of systematic reviews suggests that it would be better to frame community participation as a process supporting concrete interventions (i.e. improved birth outcomes) as evidence shows that participation is context specific.

## Introduction

The global dialogue around policies for health today places much discussion on specifically those living in poverty. Participation is not only promoted in the context of provision and utilization of health services. Advocates also highlight participation as a key factor in the wider context of importance of social determinants of health and health as a human right (WHO 2008a). Despite the growing interest in the role of participation, there is little concrete evidence that links participation directly to better health outcomes (Rifkin 2009). The absence of this link continues to be a barrier to gain full support of governments, funding agencies and health professionals to promote this approach (Atkinson *et al.* 2011). The purpose of this article is to review the research that seeks to examine the links between community participation and improve health outcomes in programmes that target poor people. To do this, it starts with systematic reviews and case studies from 2003 to 2013. Relying mostly on systematic reviews, it shows that most research studies view community participation as an intervention and use Randomized Control Trials (RCT) as the framework to investigate the link. The majority of studies find that such a link is not possible to identify because there is no standard definition of ‘community’ and ‘participation’. Where links are found, they are situation-specific and are unpredictable and not generalizable. It suggests that if community participation is viewed as a process facilitating an intervention rather than an intervention research investigating the link between participation and health status outcomes would have greater validity reflecting how intended beneficiaries see their situations rather than the views of policy makers and planners.

Participation of community members in health care is not new. An obvious example is the participation of lay/community people in the provision of care to family and community in their own cultural settings. In addition, community lay people have been involved in the delivery of allopathic health services for the last one and a half centuries. One of the most prominent experiences is the experiment of the Rockefeller Foundation in Ding Xian, China in the 1920s where local people helped deliver services in an area lacking doctors trained in Western medicine (Chiang 2001). King also records similar experiences in Africa using locals as doctor’s assistants (King 1966) in colonial Africa.

Worldwide experiences, published by the World Health Organization (WHO), argued for the importance of community participation in health care (Newell 1975). However, these experiences based on selected case studies produced assumptions rather than evidence of the value of participation. These assumptions included: (a) people will be more supportive of health services if they have been involved in decisions about how services are delivered thus promoting sustainability. (b) People will provide resources (time and money) to contribute to health improvements in their community. (c) People will change risky health behaviours when they have been involved in decisions about change. (d) People will be empowered by gaining opportunities for knowledge, skills and confidence by being involved in community health (Cueto 2004). Rifkin (2009) has analysed the consequences for building programmes on these assumptions. The results show that many publications present advocacy rather than evidence.

With the acceptance of Primary Health Care (PHC) as the official policy of the member states of WHO in the Alma Ata Declaration in 1978, the importance of community participation entered the global health policy arena. The Declaration stated that health is a human right, that the inequalities in existing health status are ‘politically, socially and economically unacceptable’ and that essential health care must be made ‘accessible to individuals and families in the community through their full participation’ (WHO 1978). The document highlighted social justice and linked it to equity and participation as principles of PHC.

Responding to the call for community participation in the Alma Ata declaration, one of the more immediate actions taken by several governments was the creation of a cadre of community health workers (CHW) to serve poor rural populations where the majority of the world’s population lived. Modelled on China’s ‘Barefoot Doctors’, they were community members trained to provide basic health care and referrals to health care centres. Embedded in the community and supported by the community, it was believed they would lower the cost of health provision. In theory, they also acted as community ‘change agents’ who would make an impact on poor health behaviours and ‘empower’ communities to make joint decisions about health care (Werner 1977). Answering Alma Ata’s call for community participation, CHWs became synonymous with PHC (Mburu 1994).

These expectations proved to be somewhat idealistic. Not only was the idea of CHWs as a means of providing a relatively cheap health service challenged but also the reality of community participation as a guarantee for uptake and support for local health services was not supported (Berman *et al.* 1987; Walt 1990). As a result, the concept of community participation became more nuanced. The argument for a wider role for community people in decisions about health programmes resulted in replacing the term ‘participation’ with ‘empowerment’ (WHO 1986). The Bamako Initiative, underpinned by the move for decentralization of health services from the centre to peripheral units identified the concerns over accountability and governance (Mehrotra and Jarret 2002). The financial crises of the 1980s added discussions about cost effectiveness and sustainability. In addition, the WHO report of the Commission on the Social Determinants of Health (2008a) and World Report on Primary Health Care (2008b) highlighted the importance of the social determinants of health and the importance of addressing issues around power and control over decisions about community health and behaviour change. These developments brought issues of empowerment, capacity building of local people, financing and programme sustainability into the dialogue.

In summary, the increasing complexity of factors influencing community participation complicated the search for a direct link between community participation and improved health outcomes. This was particularly true in the health field where the dominant paradigm, exemplified by RCTs, examines phenomena in a linear, causal relationship and explains events that do not fit into this framework as confounding variable.

## Methods

This article updates an earlier review undertaken by Rifkin (2009). It is based on a systematic search of PubMed and Google Scholar for relevant articles published between 2009 and 2012. It mainly relies upon a review of published systematic reviews in English on the topic (Table A1). Key words included community participation, CHWs, community health committees, community accountability, community engagement, participatory learning and action. Inclusion criteria were evidence of community participation in the context of health care delivery including services and promotion where health professionals have defined the community’s role. The criteria also included reviews that examined programmes where professionals designed the programme and mobilized communities to take up the benefits. This approach has been identified as ‘induced participation’ in a study by the World Bank asking ‘does participation work?’ (Mansuri and Rao 2013). It does not include research for health where remits involve communities as collaborators in research for health care improvements (Green *et al.* 2003).

## Reviews that examine the role of the community health worker

In the period that followed the Alma Ata declaration, CHW programmes proliferated. After a hiatus of interest in CHWs for about 20 years, the new concern about the crisis in human resources for health (WHO 2006) that highlighted the dismal lack of health providers especially in poor rural areas in Africa due to the HIV/AIDs epidemic resulted in the expansion of CHW programmes. Several reviews of CHW programmes have recently been published.

Bhutta *et al.* 2010 examined CHW programmes for the WHO Global Workforce Alliance. The review concentrated on the performance of CHWs to deliver credible health care interventions. However, despite the recognition that community participation was a key element of successful programmes, Bhutto and colleagues clearly state: ‘Importantly, community ownership and supervision of CHWs is a key characteristic which is insufficiently described and analyzed in available literature’ (Bhutta *et al.* 2010). In a more recent review by Perry and Zulliger (2012), the authors provide evidence of performance of CHWs and make a series of recommendations about the technical and structural support for CHWs. However, they note ‘there are very few studies that give ‘voice’ to CHWs and provide an opportunity to learn about their views regarding the challenges they face in their work and how programmes could help them to be more effective (Perry and Zulliger 2012, p. 43). These conclusions are confirmed in a review done by Naimoli *et al.* (2012) who looked at 18 programmes for the United States Agency for International Development. Their data shows that while community involvement is considered a key component of programme design there was little participation in the design, recruitment and implementation in the programmes (Naimoli *et al.* 2012). The focus on health outcomes is repeated in the Earth, Inc. Report (2012) arguing for expansion of CHWs. While noting the importance of community participation these reviews views fail to take up the challenge of examining its contribution.

### Reviews that seek evidence of a direct link between participation and improved health outcomes related to disease control and improvements in maternal and child care

Motivated by the search for replicable designs and the search for funding, researchers have increasingly sought to find evidence of a causal link between community participation and improved health status. Not surprisingly, strong efforts for the search have been made in the area of communicable disease control. For example, a systematic review of control of Chagas disease concludes that participation enhanced the control of the disease but further evidence was necessary (Abad-Franch *et al.* 2011). Concerning the examination of the detail that describes participation, the authors say

For instance, we found that most community-based experiences in Chagas disease vector control are merely, utilitarian, with little or no participation of the community in design, planning and evaluation of interventions. Effective involvement of all stakeholders along the whole process would no doubt foster true empowerment, and this could in itself result in improving health and living standards (Abad-Franch *et al.* 2011, p. 9).

No evidence is given to support this statement.

A review by Atkinson *et al.* (2011) responds to the lack of investigation into the wider role of the community by a systematic review examining communicable disease control in low- and middle-income countries using malaria as a case study. Out of 60 studies meeting criteria standards, only 4 addressed the relationship of disease transmission. The review shows that community participation has played a key role in disease control and elimination in many countries. However, the exact nature of this role is hard to define. The reason, the authors state that the potential of community participation has not been realized is that there is a lack of definitions for ‘community’ and ‘participation’ and insufficient investment in the ‘peoples component’ of the programmes.

Research undertaken at the Institute of Child Health, UK, looks at a meta-analysis of seven RCTs in Malawi, India, Bangladesh and Nepal (Prost *et al.* 2013). The intervention was using women’s groups practicing Participatory Learning and Action (PLA defined as involving the intended beneficiaries in decision making about a programme) (Rifkin and Pridmore 2001) to improve birth outcomes. Seven trials met the inclusion criteria.

Meta-analyses of all trials showed that exposure to women’s groups was associated with a 37% reduction in maternal mortality (odds ratio 0·63, 95% CI 0.32–0.94), a 23% reduction in neonatal mortality (0.77, 0.65–0.90), and a 9% non-significant reduction in stillbirths (0.91, 0.79–1.03), with high heterogeneity for maternal (I2 = 58.8%, *p* = 0.024) and neonatal results (I2 = 64.7%, *p* = 0.009). In the meta-regression analyses, the proportion of pregnant women in groups was linearly associated with reduction in both maternal and neonatal mortality (*p* = 0.026 and *p* = 0.011, respectively). A subgroup analysis of the four studies in which at least 30% of pregnant women participated in groups showed a 55% reduction in maternal mortality (0.45, 0.17–0.73) and a 33% reduction in neonatal mortality (0.67, 0.59–0.74) (Prost *et al.* 2013, p.1736)

They conclude that women’s groups are both cost-effective and a realistic way to reduce maternal deaths and improve birth outcomes rapidly and on a large scale.

Marston *et al.* (2013) investigate the effects of community participation on improving skilled care for maternal and newborn health. From the search of 11 databases with following up secondary references, they found 10 interventions. They defined interventions as getting people together to think and talk about health problems and services and having people act upon or having outsiders help people to act upon what people said. Looking at community participation as an intervention, from the evidence they state that there are few high quality quantitative studies, none of which answer the question of why interventions succeed or fail. They conclude that a qualitative research component and studies of complex interventions as part of the RCT would assess potential of generalizability and help understand the hard to measure social/political effects of participation.

Preston *et al.* (2010) examine the literature to seek evidence of the link between community participation and improvements in rural health outcomes. Of the 689 articles identified, 37 met the qualification criteria. Their review found little evidence of a direct link. However, they state lack of evidence did not mean lack of effect. They argue that it is necessary to improve our understanding about community participation in terms of the expectations of time and financing and tools to measure and understand participation in a health development context.

### Reviews that seek evidence on community participation and improved health systems including accountability

With the recognition in the 1980s that improved health status not only depended on disease control but also on the systems that delivered health care, interest began to focus on the importance of actively involving the beneficiaries of care in decisions about the provision of that care. With a focus on developing countries, Mubyazi and Hutton (2012) have reviewed the published and grey literature about community participation in the context of health planning, resource allocation and service delivery. They highlight the fact that lacking a standard definition, community participation in programmes has no common approach. Eighty-five articles met the criteria for review. Of these 37% were experimental, 55% were observational and exploratory, 42% were reviews and/or discussions. They conclude that the contribution of community participation to improving health depends on a wide variety of factors including system factors and socio-cultural factors. They point out that most authors focus on one dimension of community participation such as mechanism for community expression for public priorities. Seeing participation as a solution to one particular health problem without considering other systemic factors also limits the assessment. The review illustrates and the authors highlight that there is the lack of data about a comprehensive and generalizable approach to community participation and its relationship to improved health.

McCoy *et al.* (2011) investigate the contribution of health facility committees, a mechanism seen to give ‘voice’ to beneficiaries in the delivery of the care they receive. They also discuss the frustrations from the inability to give standard definitions of ‘community’ and ‘participation’. They identified only four cases rigorous enough to provide robust data for analysis. From this data, the authors found that it was not possible to confirm external validity. The outcomes depend on the process and the interaction between the intervention and the context.

Molyneux *et al.* (2012) review the literature examining community accountability at the peripheral health facilities. They identify 21 articles from low- and middle-income countries with robust data. The most popular mechanism for community accountability was committees (health centre and clinic, village health committees and ward committees) followed by groups, most popular women’s groups. They identify several key factors that related to strong accountability mechanisms. The success of these committees depended on how and why (political interest or response to funding) they were selected, the relationship between committees, groups and the health workers and managers and provision of support including resources by local and national governments. All these factors are processes on which community participation depends. They are context and content specific.

### Reviews that seek evidence of community participation and health promotion

Community participation, or community engagement as it is often called, has been part of the policy of the United Kingdom government since the 1970s. It is intended to involve communities in order to change poor health behaviours by involving local people, motivating better behaviour and defining how government can support their choices. In their review of this policy, Evans *et al.* (2010) note that the policy has been followed erratically over the past 40 years. They found 2155 documents. In their analysis, they highlighted the lack of RCTs available and relied on systematic reviews that used qualitative research. Only very few reviews met their quality criteria (a series of 10 questions developed by Smith *et al.* 2009) and only four reported on the process of participation and communities’ perception of quality and impact of participation. Their main finding was there was very little evidence of a direct link between participatory approaches and a ‘noteworthy’ impact on health and social outcomes.

Milton *et al.* (2011) did another study on the same topic and came to a similar but more nuanced conclusion. On the basis of 13 studies that were robust enough to meet their inclusion criteria, they found no evidence of positive impacts on population health or quality of services but found that initiatives did show a positive impact on housing, crime, social capital and community empowerment. They also point to the need for methodological developments that enable researchers to identify more robust evidence to assess multi faceted social interventions.

## Discussion

In summary, the reviews identify several common issues that challenge the investigation of a direct link between participation and improved health status. These include the lack of common definitions for the terms ‘community’ and ‘participation’, the recognition of a key role of community participation but the lack of conceptual and practical frameworks to articulate this role, and the inability to disaggregate the contribution of community participation to health from other community development improvements. The common theme is that the frameworks that have been used do not allow the results to be generalizable. Evidence shows that outcomes are determined by context and context varies. Adding a qualitative component to the research design does not address the challenge of making the findings more robust. Qualitative data only defines more clearly the importance of context and situation.

In the field of health research, intervention studies are dominant. They are designed by health professionals and seek to test a hypothesis by introducing interventions and evaluating outcomes. Based on assessments of clinical trials, the RCTs set the standard. This approach has also been used to study population health. Community participation is the intervention. The hypothesis is that this intervention will improve health outcomes. However, the evidence suggests it is not possible to adequately test this hypothesis.

Sanson-Fisher *et al.* (2007) have reviewed the complications of using RCTs for evaluating for public health outcomes. They argue that population-based interventions cannot be evaluated in this framework for a number of reasons. These include issues around population validity, time for follow-up, external validity, contamination of study population, cost, ethical and informed consent and inhibition to develop innovative research questions.

The case studies in this review all explicitly or implicitly use the RCT framework in terms of their research question. They illustrate the limitations of RCTs. Two most explicit examples are those concerning the contribution of CHWs and the systematic review of the participatory women's groups to improved birth outcomes. Concerning the former, although the reviews recognize the critical role of community participation, they focus on the causal link between service provision by CHWs and improved health status. This focus takes a mechanistic, reductionist approach to the values of CHWs. Although the reviews highlight the challenge of questions around replication, financing, sustainability and ultimately community ownership, they do not take up this challenge. Concerning the latter, as Victora (2013) discusses in the International Journal of Epidemiology, women's groups are not aimed at specific changes in health status but rather at raising the consciousness of people to take action on their impoverished lives through transforming their circumstances through action and change of power. The causal chain of poverty and transformation is not caught in a RCT.

Recognizing the limits of RCTs, important attempts have been taken to modify the approach. One example documented by the Medical Research Council in the United Kingdom (2008) recognizes ‘complex interventions’. It provides guidelines for researchers involved in non-experimental studies and interventions that go beyond delivery of health services to understand constraints on evaluation designs and to assist users of studies to assess their in terms of methodology and practical considerations. The Rockefeller Foundation of the United States has also taken up the challenge by defining and examining indicators as interventions (Davis and Kingsbury 2011). The authors argue indicators are diagnostic tools for identifying problems and needs, measuring for performance, building ways for awareness-raising and public advocacy and instruments of change. While both of these approaches seek to address concerns about the use of intervention studies they still see communities as the object not the subject of the programmes.

In the field of evaluation of public services, Pawson *et al.* (2005) have put forward the concept of realistic evaluations. They outline a step-by-step framework. Step 1: outline the theoretical framework by defining the assumptions about how the intervention(s) is seen to work and its expected impact. Step 2: look for empirical evidence to test the framework in terms of support, contradiction and/or modification. Step 3: combine the theoretical and empirical evidence and focus on the context in which the intervention(s) is applied, the mechanisms that makes it work and finally the outcomes.

Specifically in the health field, community-based participatory research and participatory research address important elements of realistic evaluation by involving community members in designing, implementing and evaluating specific health interventions. However, all these approaches are conceptualized in the context of intervention studies. Although recognizing the importance of participation as a process, to date they do not explain how these processes develop community ownership, a key challenge identified by Bhutta *et al.* (2010) in the context of CHWs. At present, health professionals make decisions about the outcomes that are to be achieved. Trickett *et al.* (2011), state this research raises the challenge that local knowledge and influence is being carried out by science devised by professionals outside the community.

Based on the findings of this article, it can be argued that a new framework is needed to understand the value and challenges of community participation to improved health outcomes. This does not suggest that an intervention research framework has failed to help us confirm the value of community participation. Many of the reviews, as noted above, have identified health improvements as a result of participation. Nor does it suggest a rejection of quantitative data to document improved health status related to community participation. Measurements are critical to confirm change and improvements. Non-Government Organizations (NGOs) and the governments of India and Brazil have national programmes where community participation is key. They have evidence of health improvements. The challenge is to define exactly how communities have benefited and why they have benefited.

In this quest, a research framework that views community participation as a process rather than an intervention is more useful. Merriam Webster defines intervention as the act or fact or a method of interfering with the outcome or course especially of a condition or process (as to prevent harm or actions or events leading to a result) (Merriam-Webster.com. 2014). It defines process as ‘actions or events leading to a result’ (Merriam Webster no date). In health improvements, an intervention is an act or method that seeks to encourage individuals and/or communities to accept a change in attitudes and behaviour to improve their health. A process is the actions over time that allow acceptance of the intervention.

A number of researchers have taken this approach to investigate participation as what supports the uptake and sustainability of a concrete intervention (an intervention which has a standard definition and a measurable outcome, e.g. improved birth outcomes). Butterfoss (2006) presents a framework to evaluate community participation as an intermediary step to health and social change. She gives tools to examine the relationship between community building and organizing principles and health outcomes. However, the evaluation framework is based on measuring participation and is, thus, a reductionist approach. It does not take into account the specific context of the process or highlight the nature of change over time. Butterfoss recognizes that measurements alone are not enough to ensure progress. Critical is how communities are defined and who represents them.

The framework most used (Molyneux *et al.* 2012, p. 3) was developed by Rifkin *et al.* (1988) and visualizes the process of community participation as a ‘spidergram’. It identifies five factors that influence community participation (needs assessment, leadership, organization, management and resource mobilization), places each on a continuum with wider participation at one end and narrow at the other, assigns a mark on the continuum for each factor, links the continua at the end of narrow participation and connects the marks. By assessing these factors at different times during a programme, planners and managers can see if participation has increased or decreased. A modification of this framework by Draper *et al.* (2010) replaces the continuum with mobilization (narrow) at one end and empowerment (wide) at the other.

This framework has been used to assess participation in relatively small health programmes. It allows programme planners and managers to document changes in community participation over time and make programme adjustments. It also allows the intended beneficiaries to express their views about participation in the community health programme and dialogue with managers about changes. Case studies using the framework include: investigating community participation in a Heart Health Program in British Columbia (Naylor *et al.* 2002); assessing rural health trusts in New Zealand (Eyre and Gauld 2003); examining CHWs in Cambodia (Jacobs and Price 2003); supporting dengue control in Cuba (Toledo *et al.* 2007): reviewing the contribution of community participation to 23 health programmes in Muldersdrift Health and Development Programme in South Africa (Barker and Klooper 2007) and assessing Safe Motherhood Health in Myanmar (Soe *et al.* 2012). The framework defines the process in specific situations related to history and culture of the community. To identify what aspects of the process might be generalizable more research is needed. This research needs to focus on the social determinants of health as discussed below.

Considering participation as a process is not merely adding a qualitative component to supporting a mixed methods approach to research or using tools to measure peoples’ behaviours and beliefs. It also includes examining the social, economic and political context over time. It includes measurements but also focuses on a holistic analysis of a specific situation. From a collection of a wider range of data, communality through comparison can be identified and the search for replicable, generalizable factors can be investigated. It is also necessary to investigate the assumptions behind the contribution and to develop frameworks for examining these assumptions. A first step is to reframe research questions to identify community participation as a process and recognize this process is a reflection of the context in which it takes place over time (Rifkin 1996). A second step is to identify and examine in detail common domains that influence these processes. Evidence is available to start this identification. Domains include leadership, capacity building, resources mobilization (internal and external) and management (inclusion of intended beneficiaries in decision making) (Rifkin *et al.* 1988; Laverack 2004; Liberato *et al.* 2011). A third step is to recognize that participation by its nature must deal with issues about power and control. Research needs to address this issue to understand the link between participation and improved health outcomes.

## Conclusion

Community participation is increasingly recognized as key to improving and maintaining interventions that improve health outcomes. To date, community participation has most often been seen as an intervention to improve health outcomes rather than a process to implement and support health programmes to sustain these outcomes. To understand the relationship between community participation and improved health outcomes, new frameworks are needed. Examining community participation as a process and dealing with critical issues around empowerment, ownership, cost-effectiveness and sustainability of health improvements would move this dialogue further.

## Appendix

**Table A1.** Table of reviews examined

Review	Purpose/aim	Key findings
Rifkin 2009	Update findings on lessons about community participation in health	- CHWs contributed to the reduction in maternal and child mortality rates
Bhutta *et al.* (2010)	Evidence of CHW programmes on Bhutta Millennium Development Goals (MDG); Maternal and child health interventions	- Decrease in the burden and costs of TB and malaria
Earth Institute Report (2012)	Scale-up and integrate CHW in the national health systems; Maternal and child health interventions	CHWs can improve health seeking behaviour and provide low-cost maternal and child health interventions; cost of CHW subsystem is estimated to be $2.62 per capita, and a programme cost of $3584 per CHW
Naimoli *et al.* (2012)	Reviews broad set of interventions; role of health systems and the community	CHWs can successfully deliver a range of preventive and curative services to improve health outcomes; - Number of factors influence CHW performance, including CHW, community characteristics; service mix, contextual factors and community are involved in CHW support activities
Perry and Zulliger (2012)	Review of literature, expert opinion;	CHWs highly effective in promoting breastfeeding; treating childhood pneumonia, diarrhoea and malaria; reducing maternal and child mortality
Abad-French *et al.* 2011	Systematic review of community participation in the control of Chagas disease	Participation enhances control of disease but further evidence is necessary
Prost *et al.* 2013	Systematic review of role of women’s participatory groups in improving birth outcomes	Women’s groups are cost-effective and improve birth outcomes rapidly on a large scale
Atkinson *et al.* 2011	Systematic review of the role of community in communicable disease control with malaria as a case study	Challenges of lack of definition for ‘community’ and ‘participation’ and poor understanding of the constructs of participation and a “peoples’ component” in control programmes
Preston *et al.* 2010	Systematic review of evidence of community participation and improvements in rural health outcomes	Lack of evidence of direct link but this did not mean lack of effect; need to improve understanding of participation in terms of time and financing and need tools to measure and understand participation
Marston *et al.* 2013	Systematic review of evidence of effects of community participation on improving skilled care for maternal and newborn health	Found very few high quality studies and none that answered question of why interventions succeed or fail; need qualitative component to study ‘complex interventions’ as part of RTCs
Mubyazi and Hutton 2012	Review of community participation in health planning, resource allocation and service delivery from published and grey literature	Barrier to evidence is lack of standard definition of ‘community’ and ‘participation’; contribution of participation depends on many factors including system factors and socio-cultural factors; lack of data to make generalizations
McCoy *et al.* 2011	Systematic review of health service committees	Barriers to evidence is lack of standard definition of ‘community’ and ‘participation’; lack of data for robust analysis; cannot confirm external validity; outcomes depend on process and context
Molyneux *et al.* 2012	Review of literature on community accountability at peripheral health facilities	Accountability depended on political interests, response to funding, selection, support from local and national government and relationships in committees and with other groups, health providers and managers
Evans *et al.* 2010	Systematic review of impact of participatory approaches on UK public health units on health and social outcomes	Little evidence of a direct link between participatory approaches and a ‘noteworthy’ impact on health and social outcomes
Milton *et al.* 2011	Systematic review of community engagement on health and social outcomes	No evidence on population health or quality of services but some positive impact on housing, crime, social capital and community empowerment

## References

[czu076-B1] Abad-Franch F, Vega MC, Rolón MS, Santos WS, Rojas de Arias A (2011). Community participation in Chagas Disease vector surveillance: systematic review. PLoS Neglected Tropical Diseases.

[czu076-B2] Atkinson J, Vallely A, Fitzgerald L, Whittaker M, Tanner M (2011). The architecture and effect of participation: a systematic review of community participation for communicable disease control and elimination. Implications for malaria elimination. Malaria Journal.

[czu076-B3] Barker M, Klopper H (2007). Community participation in primary health care projects of the Muldersdrift Health and Development Programme. Curationis.

[czu076-B4] Berman P, Gwatkin D, Burger S (1987). Community-based health workers: health start or false start?. Social Science and Medicine.

[czu076-B5] Bhutta Z, Lassi Z, Pariyo G, Huicho L (2010). Global Experience of Community Health Workers for Delivery of Health Related Millennium Development Goals: A Systematic Review, Country Case Studies, and Recommendations for Integration into National Health Systems.

[czu076-B6] Butterfoss FD (2006). Process evaluation for community participation. Annual Review of Public Health..

[czu076-B7] Chiang YC (2001). Social Engineering and the Social Sciences in China.

[czu076-B8] Cueto M (2004). The origins of primary health care and selective primary health care. American Journal of Public Health..

[czu076-B9] Davis K, Kingsbury B (2011). Indicators as interventions: pitfalls and prospects in supporting development initiatives. https://www.google.com/webhp?source=search_app#fp=cada58987153eb1f&q=Rockefeller+Foundation+wicked+indicators.

[czu076-B10] Draper AK, Hewitt G, Rifkin S (2010). Chasing the dragon: developing indicators for the assessment of community participation in health programmes. Social Science and Medicine.

[czu076-B11] Earth Institute (2012). Community Health Workers.

[czu076-B12] Evans D, Pilkington P, McEachran M (2010). Rhetoric or reality? A systematic review of the impact of participatory approaches by UK public health units on health and social outcomes. Journal of Public Health.

[czu076-B13] Eyre R, Gauld R (2003). Community participation in a rural community health trust: the case of Lawrence New Zealand. Health Promotion International.

[czu076-B14] Green LW, George MA, Daniel M, Minkler M, Wallerstein N (2003). Guidelines for participatory research in health promotion. Community-Based Participatory Research for Health.

[czu076-B15] Jacobs B, Price N (2003). Community participation in externally funded health projects: lessons from Cambodia. Health Policy and Planning.

[czu076-B16] King M (ed) (1966). Medical Care in Developing Countries.

[czu076-B17] Laverack G (2004). Health Promotion Practice: Power and Empowerment.

[czu076-B18] Liberato S, Brimblecombe J, Ritchie J, Ferguson M, Coveny J (2011). Measuring capacity building in communities: a review of the literature. BMC Public Health.

[czu076-B19] Mansuri G, Rao V (2013). Localizing Development: Does Participation Work?.

[czu076-B20] Marston C, Renedo A, McGowan CR, Portela A (2013). Effects of community participation on improving uptake of skilled care for maternal and newborn health: a systematic review. PLoS One.

[czu076-B21] Mburu FM (1994). Whither community health workers in the age of structural adjustment. Social Science and Medicine.

[czu076-B22] Medical Research Council UK (2008). Complex intervention guidance.

[czu076-B52] Merriam-Webster.com (2011). http://www.merriam-webster.com.

[czu076-B23] Mehrotra S, Jarrett S (2002). Improving basic health service delivery in low-income countries: 'voice' to the poor’. Social Science and Medicine.

[czu076-B24] McCoy D, Hall J, Ridge M (2012). A systematic review of the literature for evidence on health facility committees in low and middle income countries. Health Policy and Planning.

[czu076-B25] Milton B, Anttree P, French B (2011). The impact of community engagement on health and social outcomes: a systematic review. Community Development Journal..

[czu076-B26] Molyneux S, Atela S, Angwenyi V, Goodman C (2012). Community accountability at peripheral health facilities: a review of the empirical literature and development of a conceptual framework. Health Policy and Planning.

[czu076-B27] Mubyazi G, Hutton G (2012). Rhetoric and reality of community participation in health planning, resource allocation and service delivery: a review of the reviews, primary publications and grey literature. Rwanda Journal of Health Sciences.

[czu076-B28] Naimoli J, Frymus D, Quain E, Roseman E (2012). Community and formal health system support for enhanced Community Health Worker Performance.

[czu076-B29] Naylor PJ, Wharf-Higgins J, Blair L, Green G, O’Connor B (2002). Evaluating the participatory process in a community-based heart health project. Social Science and Medicine.

[czu076-B30] Newell K (1975). Health by the People.

[czu076-B31] Pawson R, Greenhalgh T, Harvey G, Walshe K (2005). Realistic review-a new method of systematic review designed for complex policy interventions. Journal of Health Services Research & Policy.

[czu076-B32] Perry H, Zulliger R (2012). How Effective are Community Health Workers?.

[czu076-B33] Preston R, Waugh H, Larkins S, Taylor J (2010). Community participation in rural primary health care: intervention or approach?. Australian Journal of Primary Health.

[czu076-B34] Prost A, Colbourn T, Seward N (2013). Women’s groups practising participatory learning and action to improve maternal and newborn health in low-resource settings: a systematic reviews and meta-analysis. Lancet.

[czu076-B35] Rifkin SB, Muller F, Bichmann W (1988). Primary health care: on measuring participation. Social Science and Medicine..

[czu076-B36] Rifkin SB (1996). Paradigms lost: toward a new understanding of community participation in health programs Acta Tropica.

[czu076-B37] Rifkin SB, Pridmore P (2001). Partners in Planning: Information, Participation and Empowerment.

[czu076-B38] Rifkin SB (2009). Lessons from community participation in health programs: a review of the post Alma Ata experience. International Health.

[czu076-B39] Sanson-Fisher RW, Bonevski B, Green LW, D’Este C (2007). Limitations of the randomised controlled trial in evaluating population-based health interventions. American Journal of Preventive Medicine..

[czu076-B40] Smith K, Bambra C, Joyce K (2009). Partners in health? A systematic review of the impact of organizational partnerships on public health outcomes in England between 1997 and 2008. Public Health.

[czu076-B41] Toledo ME, Vanlerberge V, Baly A (2007). Towards active community participation in dengue vector control: results from action research in Santiago de Cuba, Cuba. Transactions of the Royal Society of Tropical Medicine and Hygiene.

[czu076-B42] Trickett E, Beehler S, Deutsch C (2011). Advancing the science of community-level interventions. American Journal of Public Health..

[czu076-B43] Soe HHK, Somrongthong R, Moe S, Myint KT, Ni H (2012). Assessment of community participation in safe motherhood health education program in Shan State, Myanmar. European Journal of Scientific Research.

[czu076-B44] Walt G (1990). Community Health Workers in National Programmes: Just another Pair of Hands?.

[czu076-B45] Werner D (1977). The Village Health Worker: Lackey or Liberator?. http://www.healthwrights.org/articles/lackey_or_liberator.htm.

[czu076-B46] World Health Organization (1978). Primary Health Care.

[czu076-B47] World Health Organization (1986). Ottawa Charter on Health Promotion.

[czu076-B48] World Health Organization (2006). The World Health Report 2006: Working together for health.

[czu076-B49] World Health Organization (2008a). The Report of the Commission on the Social Determinants of Health.

[czu076-B50] World Health Organization (2008b). The World Health Report 2008: Primary Health Care: Now more than ever.

[czu076-B51] Victora C (2013). Commentary: participatory interventions reduce maternal and child mortality among the poorest, but how do they work. International Journal of Epidemiology.

